# Miniature Implantable Left Atrial/Ventricular Pump to Treat Symptoms of Heart Failure With Preserved Ejection Fraction: A Novel Device Treatment

**DOI:** 10.1016/j.jacbts.2024.10.004

**Published:** 2024-10-28

**Authors:** Andrew J. Malone, Kurdo Araz, Jemil Saidi, Donald Hickey, Darragh Colgan, Aamir Hameed

**Affiliations:** a Tissue Engineering Research Group, RCSI University of Medicine and Health Sciences, Dublin, Ireland; b Pumpinheart Ltd, Galway, Ireland; c Trinity Centre for Biomedical Engineering, Trinity College Dublin, Dublin, Ireland

No device-based therapy has been approved for use in the treatment of heart failure with preserved ejection fraction (HFpEF). It is estimated that >3 million individuals in the United States alone are affected by HFpEF, making it the most common form of heart failure and a significant public health issue.^1^ HFpEF is characterized by a reduction in left ventricular compliance, leading to an increase in left atrial (LA) pressure (LAP) and secondary pulmonary hypertension. There is a demonstrable need for a new therapy that can effectively lower LAP, ideally by aiding in the filling of the left ventricle.

The miniature implantable LA/left ventricular pump (PReduction device, Pumpinheart Ltd) described here is one such therapy. It functions by pumping just enough additional volume from the left atrium, across the mitral valve, into the left ventricle during the diastole phase of the cardiac cycle, to reduce LAP and relieve symptoms. The device is intended to be a transfemoral catheter–delivered implantable pump and motor that is electrocardiogram gated and powered by a subcutaneous implanted battery pack. The studies and data reported here were conducted on a surgically implantable version of the device.

**Figure 1:**
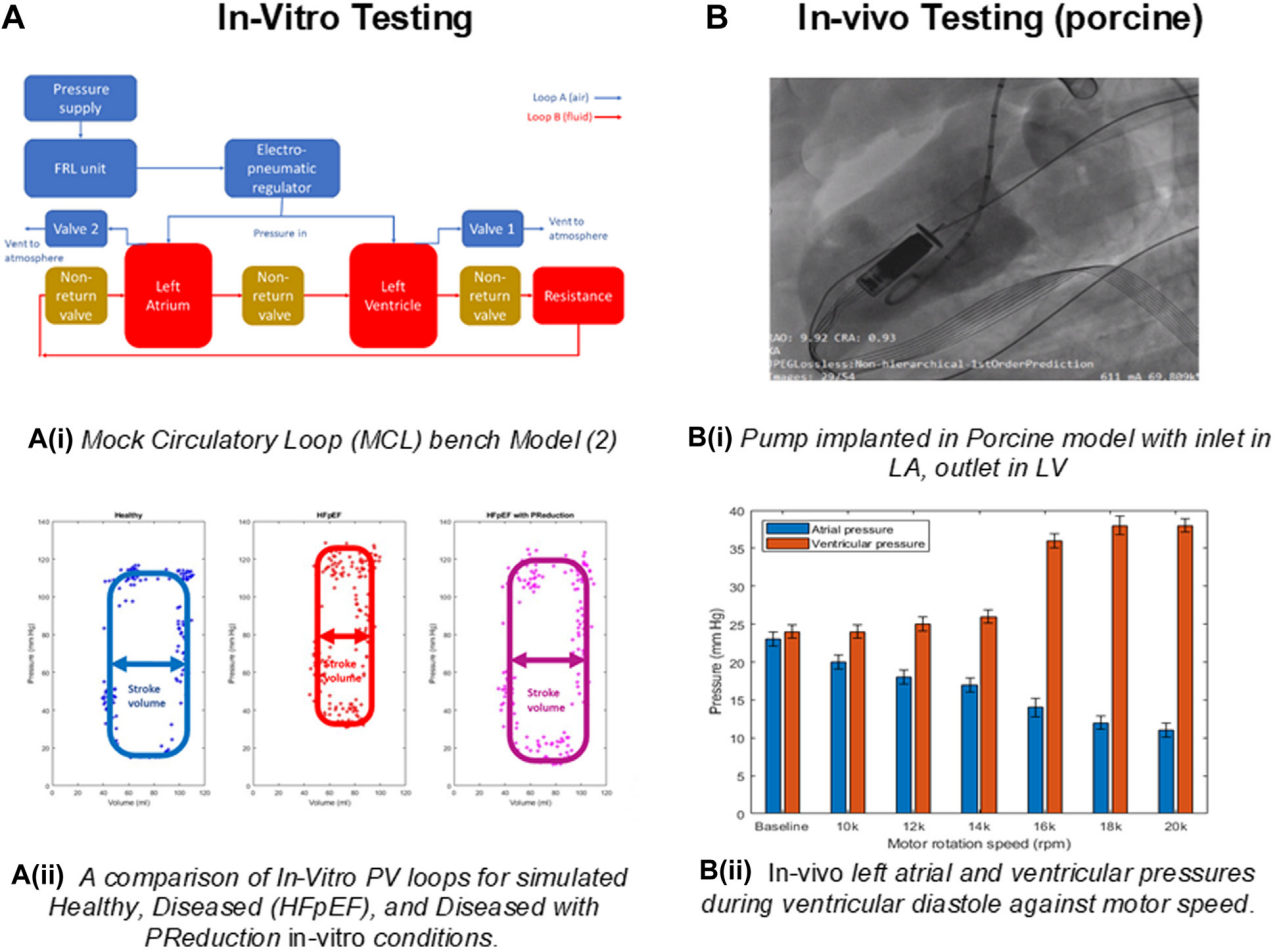
In Vitro Benchtop and Preclinical In Vivo Testing HFpEF = heart failure with preserved ejection fraction.

A robust in vitro testbed was developed by Malone et al^2^ in the form of a mock circulatory loop and is shown in Figure 1Ai. The results of the in vitro tests are shown in Figure 1Aii. Pressure-volume loops for the mock circulatory loop were generated in healthy and diseased states with the target end-diastolic pressure (EDP) increase of 15 mm Hg being met in the simulated HFpEF conditions; the recorded increase in EDP was 19 ± 4 mm Hg. The recorded stroke volume dropped from 51 ± 8 mL in the healthy state to 37 ± 11 mL in the diseased state; these findings correspond to a drop in cardiac output from 3.8 ± 0.6 L/min in a healthy state to 2.8 ± 0.8 L/min in a disease state.

When the PReduction device was inserted, EDP decreased by 18 ± 8 mm Hg, a relative reduction in the disease state of 94.7%. Stroke volume also increased to 53 ± 7 mL, corresponding to a cardiac output of 3.9 ± 0.5 L/min or a relative reduction in the disease state of 103%. This showed a return to healthy conditions with operation of the implantable pump.

In vivo testing was also completed and was performed in Veranex, Inc. This study aimed to evaluate the core system and mechanism of action of the PReduction device to treat HFpEF by using a healthy pig model. Specifically, the study evaluated the effectiveness of the PReduction device at improving the diastolic filling rate and reduction in LAP. The device was surgically inserted in the left ventricle, crossing the mitral valve and with pump inlets exposed in the left atrium (Figure 1Bi). The hypothesis was to decrease LAP by 3 to 5 mm Hg.

The baseline hemodynamic parameters were recorded with the device in situ and again at device speeds from 10,000 rpm to 20,000 rpm in 2,000 rpm intervals, with a focus on LAP and left ventricular pressure (LVP). The results of the in vivo tests are presented in Figure 1Bii. The data show bar graphs of LAP and LVP at baseline and with varying motor speeds up to 20,000 rpm. At baseline, LAP and LVP are both almost equal with no significant difference between them (*P* = 0.1202). For each motor speed, a significant difference in the pressures was found (*P* < 0.0001).

This study showed that the pump could significantly reduce LAP, consistently reducing it by >8 mm Hg above 16,000 rpm, with a corresponding increase in LVP. Furthermore, an ex vivo study completed at the LifeTec Group facility using the beating heart model equally demonstrated an LAP reduction of >8 mm Hg consistently across motor speeds above 10,000 rpm; this also corresponds to a significant increase in LVP (results not shown here).

The novel implantable device described here has exhibited early proof of mechanism of action in modulating LAP with a view to relieving the symptoms of HFpEF. This device offers the potential of a breakthrough in treating HFpEF and enabling patients to lead active lives with improved and extended quality of life.^3^ In addition, the comparatively low invasiveness of a transcatheter delivery device such as PReduction may be preferable for interventional cardiologists treating patients already weak with HFpEF.

The implantable device is currently under development, with evaluation planned for a series of acute preclinical studies, with a view to conducting feasibility, pilot, and multicenter pivotal clinical trials to assess the device’s safety and efficacy in the future.
